# Fingolimod Associated Bilateral Cystoid Macular Edema—Wait and See?

**DOI:** 10.3390/ijms17122106

**Published:** 2016-12-14

**Authors:** Refik Pul, Alma Osmanovic, Holger Schmalstieg, Amelie Pielen, Kaweh Pars, Philipp Schwenkenbecher, Kurt Wolfram Sühs, Özlem Yildiz, Benedikt Frank, Martin Stangel, Thomas Skripuletz

**Affiliations:** 1 Department of Neurology, Hannover Medical School, 30625 Hannover, Germany; osmanovic.alma@mh-hannover.de (A.O.); pars.kaweh@mh-hannover.de (K.P.); Schwenkenbecher.Philipp@MH-Hannover.de (P.S.); suehs.kurt-wolfram@mh-hannover.de (K.W.S.); yildiz.oezlem@mh-hannover.de (Ö.Y.); Stangel.Martin@mh-hannover.de (M.S.); skripuletz.thomas@mh-hannover.de (T.S.); 2 Private Ophthalmology Office, 30851 Langenhagen, Germany; info@dr-holger-schmalstieg.de; 3 Department of Ophthalmology, Hannover Medical School, 30625 Hannover, Germany; pielen.amelie@mh-hannover.de; 4 Department of Neurology, University of Duisburg-Essen, 45147 Essen, Germany; benedikt.frank@uk-essen.de

**Keywords:** multiple sclerosis, fingolimod, macular edema, VEGF, diabetes mellitus

## Abstract

Fingolimod 0.5-mg once-daily is an approved therapy for patients with relapsing–remitting multiple sclerosis (MS). Several pivotal and real-world studies have demonstrated that fingolimod is associated with the development of macular edema (ME). Herein, we present a case of a diabetic MS patient who developed severe bilateral ME during fingolimod treatment. By means of this case study we provide a detailed review about fingolimod associated macular edema (FAME), its current incidence with or without diabetes mellitus, and previous therapy attempts and outcomes in MS patients. Intravitreal administration of antibodies raised against vascular endothelial growth factor A (VEGF-A) has not yet been used in the management of FAME, however, the excellent therapeutic response in our patient may justify the use of anti-VEGF-A agents in combination with cessation of fingolimod to achieve fast resolution of FAME and to prevent visual deficits, particularly in bilateral FAME.

## 1. Introduction

Fingolimod was approved in the European Union in March 2011 as 0.5-mg once-daily therapy for patients with highly active relapsing–remitting multiple sclerosis (RRMS) [1]. It prevents the B and T lymphocyte egress from lymphoid tissues by inducing aberrant internalization of the sphingosine 1-phosphate (S1P) receptor and, thereby, reduces recirculation of autoaggressive lymphocytes to the central nervous system [2].

Originally, fingolimod was evaluated as a treatment for renal transplant rejection, wherein patients received higher doses of fingolimod (2.5 and 5 mg), in combination with cyclosporine, tacrolimus, and/or steroids [3,4,5,6]. In this context, macular edema (ME) has been identified as a specific adverse event of fingolimod in renal transplant patients [3,4,5,6]. In the phase II core study of fingolimod in multiple sclerosis (MS) and its extensions, two high doses of fingolimod (1.25 and 5.0 mg) were administered and none of the patients developed ME [7,8,9,10]. However, besides several organ-specific adverse events, ME was observed in patients receiving fingolimod during four phase III MS trials (FREEDOMS, FREEDOMSII, TRANSFORMS, and INFORMS) [11,12,13]. Since the licensing, we have treated 74 MS patients with fingolimod and one of these patients with type 1 diabetes mellitus (DM) developed bilateral diffuse cystoid ME. The aim of this study was to report the successful use of ranibizumab, an antibody raised against human vascular endothelial growth factor A (VEGF-A), in fingolimod-associated macular edema (FAME) and to provide a detailed review about this adverse event with emphasis on diabetic MS patients.

## 2. Case Presentation

A then 28-year-old Caucasian woman presented with right-sided hemihypesthesia and weakness of the right leg in March 2013. Magnetic resonance imaging showed multiple contrast-enhancing cranial and spinal lesions indicating high disease activity. Diagnosis of RRMS was made based on the revised McDonald criteria 2010 [14] supported by cerebrospinal fluid results and exclusionary laboratory tests. Prior to diagnosis, she suffered from weakness of her right leg for several weeks in 2010 and 2012. Moreover, in December 2012, she had blurred vision in the right eye for a few weeks. The only concomitant diseases were type I DM since the age of 18, treated with both long- and rapid-acting insulin (insulin glargine and aspart), and arterial hypertension, treated with enalapril 2.5 mg once daily. The patient was obese with a weight of 80 kg, height of 153 cm, and a BMI of 34.2 kg/m^2^. HBA1c values (December 2012: 9.4%, April 2013: 9.1%, July 2013: 9.8%, December 2013: 10.1%), cholesterol (December 2012: 212 mg/dL, April 2013: 301 mg/dL, July 2013: 299 mg/dL, December 2013: 210 mg/dL), as well as triglyceride levels (December 2012: 261 mg/dL, April 2013: 373 mg/dL, July 2013: 446 mg/dL, December 2013: 318 mg/dL) were markedly increased.

Intravenous steroid treatments (1 g methylprednisolone daily over five days) in March and April 2013 were considered insufficiently effective because of persisting paresis of the right leg. In May 2013, plasmapheresis was initiated and led to a slow but complete resolution of the paresis resulting in an expanded disability status scale score of 2.0 due to mild coordination deficits, reflex inequalities, absent cutaneous reflexes, and urinary urgency.

**Figure 1:**
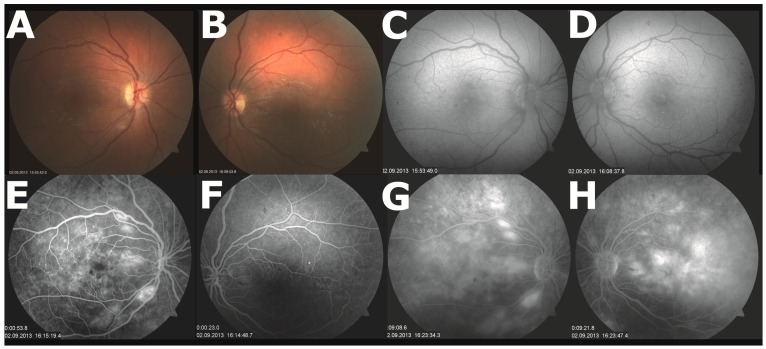
Results of the fundus photography and fluorescein angiography examinations. (**A**,**B**) Fundus photography of the right eye (**A**) revealed one small cotton-wool spot over the right inferior arcade and few intraretinal dot-and-blot hemorrhages temporal to the fovea centralis, but no hard exudates. In the left eye (**B**), it displayed few dot-and-blot hemorrhages in the superior and temporal area. Findings were in agreement with mild non-proliferative diabetic retinopathy; (**C**,**D**) Red free fundus photography allowed better visualization of the dot-and-blot hemorrhages in the right (**C**) and left (**D**) eye; (**E**,**F**) Early-phase fluorescein angiography of the right eye (**E**) showed diffuse macular hyperfluorescence and few microaneurysms. In the left eye (**F**), few microaneurysms were visible and it was not possible to assess the perifoveal arcade; (**G**,**H**) Late-phase fluorescein angiography reveals diffuse cystoid macular edema the right (**G**) and more pronounced in the left eye (**H**).

**Figure 2:**
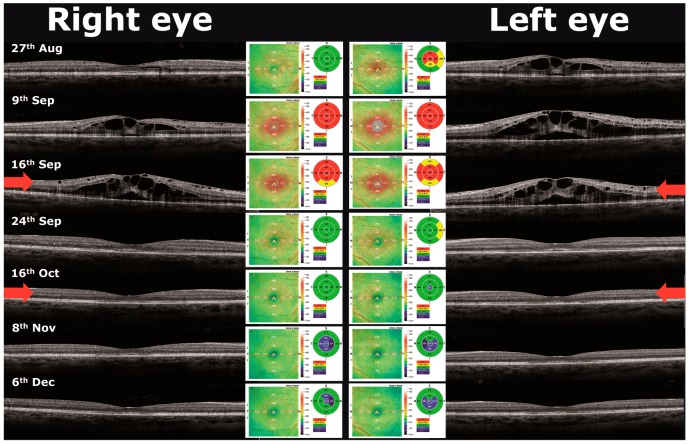
Results of the spectral-domain optical coherence tomography examinations. Spectral-domain optical coherence tomography scans through the fovea show development of severe macular edema with intraretinal und subretinal fluid and its rapid resolution after intravitreal injection of an anti-vascular endothelial growth factor (VEGF) agent into each vitreous cavity of both eyes on 16 September 2013. On 24 September 2013 optical coherence tomography (OCT) shows residual alterations of the outer retina on both eyes. Red arrows mark the intravitreal injection.

Because of the high disease activity, fingolimod was started at the end of July 2013. An ophthalmic examination before initiating fingolimod therapy was not performed. Four weeks later she complained about a progressive decrease in vision in her left eye. Best corrected visual acuity was 20/20 in her right and 20/25 in her left eye. Funduscopic examinations revealed a mild non-proliferative diabetic retinopathy in both eyes (see Figure 1A,B). Spectral domain optical coherence tomography (OCT) showed cystoid ME in the left eye with a central foveal thickness of 264 μm in the right and 463 μm in the left eye (Figure 2; 27 August 2013). Fingolimod was discontinued immediately. Two weeks later, ME was detected in both eyes, the central foveal thickness on OCT increased to 642 μm in the right and to 709 μm in the left eye and, after another week, slightly decreased to 618 μm and 648 μm in the right and left eye, respectively (Figure 2; 9 and 16 September 2013). Fluorescein angiography documented diffuse cystoid ME in both eyes, affecting the left eye more significantly (Figure 1G,H). Since the patient’s visual acuity rapidly deteriorated to 20/160 in both eyes, a volume of 0.05 mL ranibizumab (0.5 mg; Lucentis^®^) was injected into each vitreous cavity of both eyes on 16 September 2013 using a 30-gauge needle after topical anesthesia. Already after one week following administration of ranibizumab, a rapid and sustained remission of the bilateral ME was attained (Figure 2; 24 September 2013). Despite this improvement, intravitreal injection of ranibizumab into both eyes was repeated on 16 October 2013 because of bilateral central and nasal scotoma as assessed by Amsler grid testing. Best corrected visual acuity increased to 20/63 in both eyes, while treatment did not lead to any change of the scotoma. In a follow-up examination on 16 August 2015, best corrected visual acuity was 20/20 in both eyes, scotoma had significantly regressed and OCT examination did not reveal any ME (foveal thickness of 196 und 197 μm in the right and left eye, respectively).

## 3. Discussion

ME refers to accumulation of fluid in the outer plexiform and inner nuclear layers around the fovea and represents a sequel to a variety of pathologic conditions. Though it accompanies many different diseases, the disruption of the blood-retinal barrier (BRB) in perifoveal capillaries and breakdown of mechanisms that prevent the accumulation of extracellular fluid in the retina are considered to be the common cause for ME [15]. Several systemically and locally administered drugs have been implicated in the development of ME (reviewed in [15]). A possible association between fingolimod and ME has been put forward in one randomized, phase III study of renal transplant rejection even though the percentage of patients who developed ME was higher in the comparator group [5]. Three phase II/III studies have revealed that, in fact, it occurred more frequently in the fingolimod than in the comparator group [3,4,6], while no ME has been reported in two phase II studies in renal transplant patients (Table 1) [16,17]. However, there is almost no difference in the incidence of ME between the fingolimod (1.9%) and placebo/comparator group (2.0%) when dividing the number of ME cases from all renal transplant rejection studies by the total population enrolled (Table 1). However, the period of one dose-finding study [16] was rather short and regular eye examinations were performed only in one study [4]. It was speculated that the higher incidence of ME might be a result of having enrolled more subjects with risk factors (retinal vascular diseases, past ocular surgery, uveitis, DM, diabetic retinopathy) in the fingolimod group [6]. None of the renal transplantation study reports (Table 1) clarify how many patients with ME had DM. Several authors who cited these studies have claimed that patients with DM are at increased risk of developing ME when using fingolimod, although there is yet no evidence for this association [18,19,20,21,22]. This apprehensiveness probably became evident after the start of the fingolimod phase II MS study [7] because in all subsequent studies ([23], Table 2) DM was an exclusion criterion (Table 2). In phase III MS studies and their extensions, exclusion of DM as a confounding factor unveiled that, indeed, there is a dose-dependent association and ME has been reported to occur typically within three to four months after commencing fingolimod (Table 2). Overall, the incidence of ME is 0.2% when the proportion of the treatment is subtracted from that of the placebo/comparator group (0.8% minus 0.6%; Table 2) [7,12]. The selective incidence in the treatment group, on the other hand, is in line with those of one retrospective and one open-label “real-world” observation (0.9%, 3/317 [18]; 0.8%, 19/2417 [24]). However, in other studies with smaller numbers of patients no ME was observed [25,26,27] (Table 3).

Diabetic ME (DME) is one manifestation of diabetic retinopathy and a meta-analysis of 35 population-based studies worldwide suggests a considerable prevalence of 7% in the diabetic population [33]. According to this meta-analysis the prevalence of DME is more than twice as high in subjects with type 1 (14%) than in those with type 2 diabetes (6%) [33]. Several modifiable and unmodifiable risk factors, like cholesterol level [33,34], poor glycemic control [33,35], systolic blood pressure [35], presence of diabetic nephropathy [35], diabetes duration [35], higher age at onset [34], and retinal microaneurysm count [36], have been shown to influence susceptibility to the formation of ME in type 1 diabetes. Of these risk factors, we can confirm that our patient had a poor glycemic and cholesterol/triglyceride control, increased systolic blood pressure values, and a higher age at onset of type 1 diabetes (cumulative incidence of 34% at onset group 15–40 years [34]).

In our patient, OCT scans of both macular regions did not reveal any microaneurysm that could be attributed as a potential leakage source, suggesting a generalized breakdown of the BRB. The diffuse cystoid occurrence accompanied by neurosensory retinal detachment is common in DME, but uncommon in eyes with mild, non-proliferative diabetic retinopathy [37]. The immediate onset of bilateral ME after commencing treatment with fingolimod points to an association with this treatment. DME constitutes a chronic disease subject to recurrences and, in general, requires longer treatment with ranibizumab [38,39]. Complete and long-term restoration of macular thickness and/or visual acuity after only two injections of ranibizumab is fairly unlikely for DME and this therefore represents the main argument that our patient experienced FAME. FAME has been reported by more than a dozen case studies (Table 4) and it should be stressed that the typical pattern of macular edema evident in the fluorescein angiogram typically displays focal (perifoveal) retinal capillary dye leakage [40,41,42,43,44,45].

So far, only two cases of ME in diabetic MS subjects following fingolimod treatment have been reported in case studies (Table 4). The mean time of occurrence seems to be shorter in these patients (0.5 ± 0.4 months; *n* = 3 including our case) as compared to non-diabetic MS patients (2.8 ± 0.5 months), but the number of diabetic cases is too low to perform statistical analysis. All diabetic MS patients developed bilateral ME speculating that retinal alterations due to diabetic retinopathy may be the cause for this symmetric occurrence. Apart from these single case reports, and in contrast to the clinical development studies, MS patients with controlled DM were included in real-world observations (Table 3). Of these observations, only two studies present the numbers of diabetic MS patients and of those who have suffered from ME [18,24]. Based on the total number of diabetic MS patients from these studies there is an incidence of 5.3%, which is approximately seven times higher than in non-diabetic patients. However, none of these studies stated the presence and/or degree of retinopathy, duration and type of DM, and whether ME occurred on a bi- or unilateral basis. The incidence data of ME in diabetic patients without MS vary from 0% (3 years follow-up) up to 26.1% (25 years follow-up) [40], suggesting an actually higher incidence in fingolimod-treated diabetic MS subjects.

The efficacy of fingolimod is mainly reliant on its phosphate ester metabolite, which acts as a high-affinity ligand for the G-protein-coupled receptors S1P_1_ and S1P_3–5_ with the highest affinity for the S1P_1_ receptor [41,42]. For the S1P_1_ receptor, it has been shown that fingolimod phosphate initially operates as a functional agonist and overstimulates it, which causes endocytic internalization and the uncoupling of the receptor from its G protein receptor by the recruitment of β-arrestins to the receptor complex [41]. In the long run, fingolimod acts as a functional antagonist because its sustained exposure causes a reduction in the S1P_1_ receptor numbers on the cell surface [42,43]. In vitro studies have shown that this reduction persists even after discontinuation of treatment, suggesting a disturbed recycling of the receptor back to the plasma membrane, for example, by repeated dissociation-association cycles of fingolimod phosphate [41,44]. The S1P_1_ receptor, which is highly expressed on endothelial cells, has been implicated in the regulation of the vascular endothelial barrier function by cell cytoskeleton rearrangement and assembly of intercellular junctions [45,56,57]. Several in vitro and rodent studies have clearly demonstrated that fingolimod promotes tightening of the endothelial cell barrier [58,59,60,61]. All these studies, however, have in common that fingolimod was not administered continuously [58,59,60] or given over a short period [61], suggesting that their results most probably reflect the initial stimulation of the S1P_1_ receptor. Owing to the fact that continuous treatment with fingolimod decreases surface expression of the S1P_1_ receptor and that S1P_2_/S1P_3_ activation increases endothelial barrier permeability [62], one possible explanation for the development of ME might be a shift of the balance from S1P_1_ towards S1P_2_/S1P_3_ signaling resulting in the activation of Rho/ROCK signaling pathways [62]. However, in one recent study, attenuation of retinal vascular leakages after a 12-week fingolimod treatment in streptozotocin (STZ)-induced diabetic rats has been reported [63]. Moreover, there are several works underlining that long-term treatment with fingolimod does not impair endothelial barrier function [64,65], which might be explained by the fact that, despite membrane downregulation, the internalized S1P_1_ receptor still actuates signaling pathways [66] or that this function does not depend on the S1P_1_ receptor at all [67]. Conversely, it has been shown that long-term treatment with fingolimod worsens vascular leakage in bleomycin-induced lung injury [68]. Thus, it remains completely unclear how fingolimod might trigger ME.

VEGF-A, known to increase microvascular permeability, is one of the major contributors to the development of ME and is markedly elevated in patients with diabetic retinopathy [69]. In the literature, there are different results regarding the expression of VEGF-A under fingolimod therapy. Decreased VEGF-A expression/secretion has been reported in cholangiocarcinoma [70] and decidual natural killer cells [71], in lung injury [72], and renal fibrosis model [64]. Fingolimod did not alter VEGF-A expression in chondrocytes [73]. Following fingolimod treatment, increased VEGF-A expression/secretion has been observed in the periinfarct cortex after photothrombotic stroke [74] and in Jurkat cells [75]. These disparate results may reflect concentration-depending effects or be based upon different signaling pathways depending on the target tissue/cell. To our knowledge, there is currently no study that examined direct effects of fingolimod on endothelial VEGF-A secretion.

Intraocular injection of VEGF-neutralizing antibodies is a first-line treatment for DME, but it has hitherto not been used in the management of FAME. Anti-inflammatory topical medications, such as prostaglandin synthesis inhibitors (nepafenac, bromfenac, ketorelac), carbonic anhydrase inhibitor (acetazolamide), and steroids (prednisolone, dexamethasone, difluprednate), have been reported to improve resolution of ME (Table 4). For the efficacy of those topical medications, discontinuation of fingolimod seems to be a critical step, as otherwise ME may remain unresolved [22], prolong its resolution [54], recur [50], or be associated with other eye complications [51,54]. In more severe ME cases, some patients experienced only partial [22,49,51] or no recovery [55] by topical medications. Notably, some of these patients were treated successfully with intravitreous [55] or subtenon injection of a synthetic steroid (triamcinolone) [51] as a second-line therapy. Similar to our case, the intravitreal injection particularly resulted in hastened resolution of ME [55] as compared to a mere discontinuation of fingolimod or treatment with topical drugs. Such a rapid resolution is exactly required in bilateral ME because the “wait-and-see” strategy may lead to longer periods of incapacity for work and might result in residual visual deficits. We preferred the anti-VEGF therapy in order to avoid steroid-related adverse events, such as ocular hypertension, glaucoma, and cataract formation. The intraocular injection of this agent was well tolerated by our patient and ME resolved already after one injection. A second injection was given due to the presence of scotoma which, interestingly, did not respond to this treatment as quickly as the ME did, but regressed considerably within a few weeks. However, our patient still exhibits small scotomas in the central field of both eyes and we think that earlier treatment may have prevented them.

In conclusion, for the approved fingolimod dose, the current incidence of FAME is 0.2%. Real-world observations point to a higher incidence of FAME in diabetic MS subjects, but their numbers are too low to provide accurate incidence data. FAME in diabetic MS patients seems to occur earlier and is more frequently associated with bilateral occurrence. Bilateral occurrence of ME is an extensively debilitating condition that warrants early and effective treatment. Discontinuation of fingolimod in combination with intraocular injection of anti-VEGF-A agents may provide fast resolution of ME and prevent visual deficits. More data about diabetic MS patients treated with fingolimod are required to estimate accurate incidence data, identify factors that militate against fingolimod treatment, and to establish a treatment guideline for the management of ME. Future research is required to elucidate whether fingolimod modulates the expression of endothelial VEGF-A and whether increased VEGF-A serum levels predispose for the development of ME. Hitherto existing data indicate that diabetes mellitus is a relative contraindication for fingolimod treatment. Anti-VEGF drugs need to be evaluated in nondiabetic patients in order to gain certainty of their general efficacy in FAME.

## Figures and Tables

**Table 1:** Frequency of macular edema in clinical trials that evaluated efficacy of fingolimod in renal transplant rejection. * This study was a dose-finding study, wherein, in addition to 0.5 mg fingolimod, doses of 0.25 (*n* = 43), 0.5 (*n* = 43), and 1.0 mg (*n* = 40) of fingolimod were also administered to study participants. No macular edema was observed at any of these dosages.

Study	Phase	Fingolimod 2.5 mg % (*n*)	Fingolimod 5 mg % (*n*)	Placebo or Active Comparator	Combination	Exposition Time
Tedesco-Silva et al. 2004 [16] *	II	0% (0/41)	-	0% (0/41)	Cyclosporine + Steroids	3 months
Tedesco-Silva et al. 2006 [3]	III	1.8% (4/224)	3.4% (8/234)	1.3% (3/229)	Cyclosporine + Steroids	12 months
Salvadori et al. 2006 [5]	III	1.3% (3/219)	2.2% (5/224)	3% (6/226)	Cyclosporine + Steroids	12 months
Mulgaonkar et al. 2006 [17]	II	0% (0/150)	0% (0/72)	0% (0/39)	Cyclosporine + Steroids	12 months
Tedesco-Silva et al. 2007 [4]	II	1.1% (1/89)	0% (0/87)	0% (0/94)	Cyclosporine + Steroids	12 months
Hoitsma et al. 2011 [6]	III	12.2% (6/48)	-	9.3% (5/54)	Tacrolimus + Steroids	12 months
Summary		1.8% (14/771)	2.1% (13/617)	2.0% (14/683)	-	-
		1.9% (27/1388)		-	-	-

**Table 2:** Frequency of macular edema in multiple sclerosis clinical trials. Percentage incidence was calculated by dividing the number of macula edema cases in the study by the total population enrolled in the reviewed studies. * One case of macular edema has been reported in the continuous fingolimod group but without specifying the fingolimod dosage. n.a. = not applicable.

Study	Phase	Acronym	Description	Fingolimod 0.5 mg % (n)	Fingolimod 1.25 mg % (n)	Placebo or Active Comparator	Exposition Time
Kappos et al. 2010 [13] Kappos et al. 2015 [28]	III Extension	FREEDOMS	Core study Placebo-fingolimod Continuous group	0% (0/425) 0.6% (1/155) 0.3% (1/331)	1.6% (7/429) 0% (0/145) 0.3% (1/289)	0% (0/428) n.a. n.a.	24 months up to 52 months
Calabresi et al. 2014 [11]	III	FREEDOMSII	Core study	0.8% (3/358)	1.1% (4/370)	1.2% (6/487)	24 months
Cohen et al. 2010 [12] Khatri et al. 2011 [29] Cohen et al. 2016 [30]	III Extension Extension	TRANSFORMS	Core study Placebo-fingolimod Continuous group Continuous group	0.5% (2/429) 0.6% (1/167) *% (*/356) 0% (0/404)	1% (4/420) 1.1% (2/174) *% (*/330) 0% (0/368)	0% (0/431) n.a. n.a. n.a.	12 months 24 months up to 54 months
Saida et al. 2012 [23] Kira et al. 2014 [31]	II Extension	-	Core study Placebo-fingolimod Continuous group	0% (0/57) 0% (0/27) 0% (0/47)	0% (0/57) 0% (0/27) 0% (0/46)	0% (0/57) n.a. n.a.	6 months 12 months
Lublin et al. 2016 [32]	III	INFORMS	Core study	2.1% (7/336)	switch to 0.5 mg due to protocol amendment on 19 November 2009	1.2% (6/487)	36–60 months
Summary				0.8% (15/1954)	1.1% (18/1622)	0.6% (12/1890)	-
				0.9% (33/3576)		-	

**Table 3:** Frequency of macular edema (ME) cases in real-world multiple sclerosis (MS) populations. n.m. = not mentioned; n.a. = not applicable.

Study	*n*	Study Type	Study Duration/Follow-up Time	ME Cases (*n*)	*n* Diabetic MS Patients	*n* ME in Diabetic MS Patients
Ontenada et al. 2012 [18]	317	Retrospective	3 months	3	12	1
Gold et al. 2014 [24]	2417	Open-label	4 months	19	26	1
Al-Hashel et al. 2014 [27]	175	Retrospective	Up to 22 months	0	n.m.	n.a.
Ordonẽz-Boschetti et al. 2015 [26]	138	Open-label	4 months	0	n.m.	n.a.
Correia et al. 2016 [25]	104	Retrospective	Up to 21 months	0	n.m.	n.a.

**Table 4:** Case studies of fingolimod associated macular edema in multiple sclerosis (MS) patients * except one subject who was a renal transplant recipient in a phase IIIb clinical trial of fingolimod. M = male; F = female; U = unilateral; B = bilateral; ST = subtenon; IV = intravitreal; R = resolved; NR = not resolved; NM = not mentioned.

Study	Age	Gender	Diabetes/Uveitis	Onset (Months)	Time of Resolution (Months)	Uni- or Bilateral	Fingolimod	Therapy (*Outcome*)
Saab et al. 2008 * [46]	58	F	No	3.0	2.0	U	Discontinued	-
Turaka and Bryan 2012 [47]	52	M	No	3.0	3.0	U	Discontinued	Prednisolone
Liu and Cuthbertson 2012 [48]	34	F	No	0.2	NR.	B	Discontinued	Ketorolac and prednisolone (*persistence of minimal ME in the right eye after 5 months*)
Afshar et al. 2013 [49]	52	M	No	0.2	1.0	U	Continued	Nepafenac and prednisolone
	60	F	Diabetes	0.3	1.0	B	Discontinued	-
	57	M	No	1.0	NR	U	Discontinued	Bromfenac (*epiretinal membrane and still increased foveal thickness after 3 months*)
Chui et al. 2013 [50]	67	F	No	6.0	1.2	U	Discontinued	Ketorolac and dexamethasone
Minuk et al. 2013 [51]	58	F	Uveitis	2.0	1.0	B	Discontinued	Ketorolac, prednisolone, ST triamcinolone injection
Coppes et al. 2013 [21]	60	F	Diabetes	0.3	1.6	B	Discontinued	-
Li et al. 2014 [22]	37	F	No	4.0	NR	U	Continued	- (*not resolved after 25 months*)
Kim et al. 2015 [52]	62	F	No	2.5	1.0	B	Discontinued	Ketorolac and prednisolone
Ueda and Saida 2015 [53]	31	M	No	1.0	1.0	U	Discontinued	Betamethasone (*resolved in 1 month but persistence of decreased visual acuity*)
Schröder et al. 2015 [54]	24	F	No	1.0	NM	B	Discontinued	Acetazolamide (*visual acuity returned to normal after 2 weeks while it remains unknown whether ME resolved*)
Thoo et al. 2015 [55]	59	F	No	0.7	1.0	B	Continued	Prednisolone and IV triamcinolone
	66	F	No	12.0	2.0	U	Continued	Keterolac and prednisolone IV triamcinolone (*resolved in 1 week after IV triamcinolone*)
